# Advanced Extraction Techniques for Bioactive Compounds from Berry Fruits: Enhancing Functional Food Applications

**DOI:** 10.3390/foods13244115

**Published:** 2024-12-19

**Authors:** Aneta Krakowska-Sieprawska, Justyna Walczak-Skierska, Paweł Pomastowski, Róża Sobolewska, Jarosław Głogowski, Cezary Bernat, Katarzyna Rafińska

**Affiliations:** 1 Interdisciplinary Centre of Modern Technologies, Nicolaus Copernicus University, Wileńska 4 St., 87-100 Torun, Poland; walczak-skierska@umk.pl (J.W.-S.); p.pomastowski@umk.pl (P.P.); 2 Fortuna Company, Tymienice 88, 98-220 Zduńska Wola, Poland; roza.sobolewska@fortuna.com.pl (R.S.); jaroslaw.glogowski@fortuna.com.pl (J.G.); cezary.bernat@fortuna.com.pl (C.B.); 3 Department of Environmental Chemistry and Bioanalytics, Faculty of Chemistry, Nicolaus Copernicus University, Gagarina 7 St., 87-100 Torun, Poland

**Keywords:** supercritical fluid extraction, accelerated solvent extraction, bioactive compounds, antioxidant activity, plant material processing methods

## Abstract

The modern functional food market is developing dynamically, responding to the growing demand for products combining nutritional and health-promoting values. At the center of this evolution are natural bio-organic extracts, rich in bioactive compounds such as antioxidants, polyphenols, flavonoids, carotenoids, and vitamins, which can enrich traditional food products, including fruit juices, increasing their health-promoting values. The aim of the research was to compare the efficiency of extraction of bioactive compounds from various forms of plant raw material (dried, freeze-dried, frozen material) using innovative techniques: supercritical fluid extraction (SFE) and accelerated solvent extraction (ASE). The research showed that the ASE method demonstrated higher extraction efficiency, in some cases exceeding 40%, whereas SFE exhibited superior selectivity, achieving higher carotenoid contents (105.59 mg/100 g in sea buckthorn powder) and antioxidant activity (234.67 µmol TEAC/g in black elderberry fruit). The use of advanced extraction techniques is a modern approach to juice production, in line with current trends in functional food and healthy eating, which can contribute to the prevention of lifestyle diseases.

## 1. Introduction

The contemporary functional food market is experiencing dynamic growth, responding to the increasing consumer demand for products that combine nutritional and health-promoting properties. At the center of this evolution are plant-derived bioactive extracts, rich in bioactive compounds such as antioxidants, polyphenols, flavonoids, carotenoids, and vitamins, which can be used to enrich traditional food industry products, including fruit juices, thereby enhancing their health-promoting properties. This approach aims to improve and enhance the characteristics of the input product, i.e., strengthening antioxidant properties, extending the shelf life of the product (natural preservatives), and improving taste and color (natural dyes) [1,2]. The development of extraction techniques is an important task in the selective isolation of biologically active compounds from plant material.

The use of advanced extraction techniques to obtain bioactive compounds from plant materials represents a modern approach to juice production, fitting into current development trends in the field of functional food [3]. This trend fits into the broader context of growing consumer awareness of healthy eating. Although knowledge of the term “functional food” among Polish consumers is still limited, research indicates an increasing interest in products with health-promoting properties. The development of the functional food market, including innovative juices enriched with natural extracts, responds to these changing consumer preferences and opens new perspectives for the food industry. The increasing global demand for functional food products enriched with natural extracts is driven by rising consumer interest in health and wellness. This trend reflects growing health awareness, the desire for preventive healthcare, and lifestyle shifts influenced by urbanization and aging populations. Key factors include consumer interest in food products that not only satisfy basic nutritional needs but also offer additional health benefits, such as improved immunity, better digestion, or enhanced cognitive function [4]. While extensive research has been conducted on pomace, limited studies directly compare the complementary advantages of accelerated solvent extraction (ASE) and supercritical fluid extraction (SFE) in extracting diverse bioactive compounds, particularly for industrial scalability.

The extraction of bioactive compounds from various plant materials, such as sea buckthorn, rowan, wild rose, or chokeberry, is a complex process that requires the application of advanced techniques. The ultimate goal is to isolate the desired chemical substances from plants using a solvent. Traditional extraction methods, such as maceration and Soxhlet extraction, were not employed in this study due to their limitations, including longer processing times, higher energy consumption, and extensive use of organic solvents, which pose environmental and safety concerns. Innovative extraction techniques, such as SFE and ASE, open new possibilities for isolating valuable secondary metabolites while preserving their original structure and biological properties [5,6]. The ASE uses high pressure and temperature to achieve rapid extraction, while SFE operates under supercritical conditions, enabling selective extraction of low-polarity compounds with minimal solvent usage. Both techniques align with the principles of green chemistry and enable the production of extracts with a high content of bioactive compounds while minimizing the use of harmful organic solvents [6,7]. While numerous studies have focused on individual extraction techniques, there is limited comparative research evaluating ASE and SFE for their complementary advantages in isolating bioactive compounds from berry fruits. This study aims to bridge this gap by evaluating the efficiency, selectivity, and antioxidant activity of extracts obtained using these methods.

The inspiration for using extracts from berry plants with health-promoting properties in the production of innovative juices comes from both scientific knowledge and folk traditions, which are an integral part of our culture. This approach not only enhances the nutritional value of products but also may contribute to the prevention of neurodegenerative and lifestyle-related diseases, including cancer, type 2 diabetes, obesity, and cardiovascular diseases [8,9]. By employing two complementary extraction methods, it is possible to obtain bioactive compounds with different physicochemical properties. The use of innovative extraction techniques allows for the isolation of polyphenolic compounds and carotenoids as health-promoting components of the juice. Their high content in the extract may help mitigate reactive oxygen species in the body. Due to their unique antioxidant properties, these extracts could serve as natural preservatives for a new generation of juices, enabling a reduction in preservation parameters, including pasteurization [4]. Enriching juice with extracts from wild rose, similar to those from sea buckthorn, will increase the level of vitamin C. Furthermore, literature data indicate that wild rose extract inhibits browning and ensures color stability in juice [10]. An extract obtained from rowan is unique due to its proven medicinal properties, such as its antidepressant effects, and its high content of vitamin A (retinol), beta-carotene, vitamin C, and vitamin K, which collectively contribute to its beneficial effects on gastrointestinal health [11]. Chokeberry, like black elderberry, is classified as a “superfruit” because it is a unique source of antioxidants that inhibit aging processes and positively impact the cardiovascular system [12]. In the case of plant materials, the ASE technique is aimed at obtaining natural dyes. These dyes, due to their unique chemical structure containing magnesium, can act as natural antioxidants and modulators of the nervous system, which is significant in the context of functional food production [13].

The aim of the research was to compare the efficiency of extracting bioactive compounds from various forms of plant raw material (dried, lyophilized, and frozen material) using complementary techniques: supercritical fluid extraction (SFE) and accelerated solvent extraction (ASE). The raw materials (sea buckthorn, chokeberry, black elderberry, rowan, wild blueberry, and wild rose) were selected based on ethnopharmacological premises, which contribute to the development of innovative food products falling into the category of functional food.

## 2. Materials and Methods

### 2.1. Plant Material

The plant materials used in the study included the following:

- Sea buckthorn (*Hippophae rhamnoides* L.)
- Chokeberry (*Aronia melanocarpa* (Michx.) Elliott)
- Black elderberry (*Sambucus nigra* L.)
- Rowan (*Sorbus aucuparia* L.)
- Wild blueberry (*Vaccinium myrtillus* L.)
- Wild rose (*Rosa canina* L.)

Table 1 presents the selected plant materials that were used in the studies on the content of bioactive compounds.

### 2.2. Extraction Methods

Supercritical fluid extraction (SFE) was carried out using a MV-10 ASFE system (Waters Corp., Milford, MA, USA) equipped with a 10 mL extraction cell. Approximately 1.5 g of plant material was loaded into the extraction cell, which was packed with glass beads. Preliminary SFE experiments were conducted to determine the target compounds. Extractions were performed at 60 °C and 200 bar, with a 30 min static time and a 30 min dynamic mode (continuous flow), using scCO_2_ as the solvent and 96% EtOH as a co-solvent. The flow rates were 10 mL/min for scCO_2_ and 0.4 mL/min for 96% EtOH. The obtained extracts were stored in a refrigerator until further analysis.

Accelerated solvent extraction (ASE) was conducted using the Dionex ASE 350 system (Thermo Scientific, Waltham, MA, USA). Approximately 1.5 g of plant material was mixed with glass beads and loaded into the extraction cell. The extraction was performed under the following conditions: 96% ethanol (EtOH) as the solvent, a temperature of 60 °C, a pressure of 10 MPa, a static time of 15 min, and two static cycles. The obtained extracts were stored in a refrigerator until further analysis.

The extraction parameters were selected based on preliminary optimization experiments and established literature findings, ensuring the effective extraction of thermally sensitive bioactive compounds, such as polyphenols and carotenoids, while maximizing yield and maintaining stability.

### 2.3. Determination of Dry Matter Content (DM)

To determine the extraction efficiency of the extraction process, the dry mass of the extracts was first obtained. To do this, 1 mL of SFE and ASE extract was measured into Eppendorf tubes and evaporated. The procedure was carried out for each extract in three repetitions. The extraction efficiency was calculated using the following relationship:

(1) $$Y_{extract}(\%)=\frac{\;m_{extract}}{m_{feed}}\;\cdot 100$$

where *Y_extract_* is extraction yield expressed in %, *m_extract_* is the dry extract mass (g), and *m_feed_* is the feed mass (g).

### 2.4. Determination of Total Phenolic Content (TPC)

The total content of phenolic compounds in the obtained extracts was determined using the Folin–Ciocalteu (FC) method. The procedure was based on the method of Singleton et al., with modifications [14]. To 12 μL of the extract, 188 μL of deionized water and 12 μL of Folin–Ciocalteu reagent were added. The mixture was incubated in the dark for 8 min, and then 38 μL of 20% sodium carbonate was added. After 30 min of incubation at 20 °C in the dark, the absorbance was measured at a wavelength of 765 nm using a Varioskan™ LUX multimode microplate reader (Thermo Fisher Scientific, Waltham, MA, USA). The measurement was performed against a prepared blank sample. The results were expressed as gallic acid equivalents (GAE) in milligrams per gram of dry extract.

### 2.5. Determination of Total Carotenoids and β-Carotene Content

The determination of total carotenoids was carried out according to the Polish Standard PN-90/A-75101/12 with some modifications [15]. Specifically, the sample was prepared by combining 3 mL of the extract with 1 mL of ethyl ether. Carotenoids extracted from plant material should be obtained using a mixture of ethyl ether and ethanol in a ratio of 5/10–15 (*v*/*v*). After extraction, the obtained extract was transferred to 96-well plates in 250 μL aliquots. Then, a spectrophotometric measurement was performed, measuring the absorbance of the extract at a wavelength of 467 nm, which corresponds to the absorption maximum of β-carotene. The calculation of carotenoid concentration, expressed as β-carotene, was performed using the equation:

A = 0.46605n + 0.0332 [μg/mL](2)

where A represents the concentration of β-carotene in 1 mL of the solution [µg/mL], and n is the absorbance at 467 nm.

Then, the total carotenoid content in the sample was calculated according to the formula:

X = (A × V × 100)/(G × 100) [mg/100g] (3)

where A is the concentration of β-carotene in 1 mL of the solution [µg/mL], V is the total volume of the extract in mL, and G is the sample mass in grams.

### 2.6. DPPH Method

The study assessed the free radical scavenging activity of the obtained extracts using the DPPH (2,2-diphenyl-1-picrylhydrazyl) radical scavenging method. The procedure was based on the method described by Espín et al., with some modifications [16]. To 200 μL of a 0.1 mM ethanol solution of DPPH (initial absorbance of DPPH = 0.9), 50 μL of the extracts were added and incubated in the dark for 30 min. Absorbance was measured at a wavelength of 517 nm using a Varioskan™ LUX Multimode microplate reader (Thermo Fisher Scientific, Waltham, MA, USA). The results were expressed as micromoles of Trolox Equivalent Antioxidant Capacity (TEAC) per gram of dry extract.

### 2.7. Determination of Phenolic Compounds Using HPLC-ESI-MS/MS Analysis

Phenolic compounds were identified using a method developed by Krakowska et al. [6]. For the analysis, 1 mL of the extracted sample was evaporated, and the dry residue was reconstituted in 1 mL of methanol before filtration. The analysis was conducted using a Shimadzu LC-MS 8050 triple quadrupole mass spectrometer (Kyoto, Japan), which included a binary solvent delivery system (LC-30 CE), a controller (CBM 20 A), an autosampler (SIL-30 A), and a column thermostat (CTO-20 AC). Data analysis was performed with the LabSolution 5.8 software. Separation of phenolic compounds was achieved on a Kinetex F5 column (100 × 2.1 mm, 2.6 μm; Phenomenex, Torrance, CA, USA), using 0.1% formic acid in water as mobile phase A and acetonitrile as mobile phase B. The gradient program was as follows: 0–7 min, 0–80% B; 7–8 min, 80% B; AND 8–10 min, 80–0% B. The flow rate was set at 0.4 mL/min, with an injection volume of 10 μL. The MS/MS analysis was performed in both positive and negative ionization modes. Multiple reaction monitoring (MRM) was employed for qualitative and quantitative analysis. Key electrospray ionization (ESI) settings were as follows: nebulizing gas flow at 3 L/min, heating gas flow at 10 L/min, drying gas temperature at 400 °C, desolvation line (DL) temperature at 250 °C, and interface temperature at 300 °C. Detailed MRM transitions for all identified compounds were reported by Krakowska et al. [6].

### 2.8. MALDI-TOF-MS Analysis

For the MALDI-TOF-MS analysis, 1 μL of ethanol extract obtained through ASE and SFE methods was deposited onto a ground steel MALDI target. The analysis was performed using an UltrafleXtreme II MALDI-TOF/TOF mass spectrometer (Bruker Daltonics, Bremen, Germany), equipped with a modified neodymium-doped yttrium aluminum garnet (Nd) laser (smartbeam II) operating at a frequency of 2 kHz and a wavelength of 355 nm. Spectra were recorded in reflector positive mode, with an acceleration voltage of 25 kV, covering an *m*/*z* range of 100–4500 Da. All acquired mass spectra were processed using the flexControl and flexAnalysis 3.4 software (Bruker Daltonik). Additionally, cluster analysis was conducted using ClinProTools 3.4 software (Bruker Daltonik) to further process the MALDI-TOF/TOF MS data.

## 3. Results and Discussion

Research on the extraction of bioactive compounds from plants focuses on the efficiency of various methods, which is crucial for the food industry. In particular, SFE and ASE are two innovative techniques that differ in terms of efficiency, quality of obtained extracts, and the type of bioactive compounds recovered. The present study focused on comparing these two methods in the context of phenolic content, carotenoid content, and antioxidant activity in various plant samples.

### 3.1. Comparison of Extraction Methods

The results indicate significant differences in the efficiency of both methods:

- Yields of Extraction

The extraction efficiency was significantly higher for the ASE method, both for freeze-dried, dried, and frozen materials. The highest values, exceeding 40%, were obtained for sea buckthorn fruit halves and the powder and grits from chokeberry. The SFE extraction method demonstrated a considerably lower extraction efficiency, which exceeded 10% only for freeze-dried sea buckthorn halves and frozen wild berry fruits. Such differences indicate a greater effectiveness of ASE in obtaining bioactive compounds, which can be advantageous in an industrial context where efficiency is a key factor. However, as noted by Patra et al. [17], higher efficiency does not always translate to extract quality, suggesting that some bioactive compounds may be better preserved during the SFE process. Previous studies using MALDI-TOF-MS have shown that SFE is a more selective extraction method than ASE, as ASE extracts contain a significant amount of interfering compounds, such as peptides and small proteins, as well as fragments of polysaccharides [7]. A high content of interfering compounds in the extract can negatively affect the stability and turbidity of juices, ultimately reducing their final quality. It is also worth noting that differences in efficiency may result from various extraction parameters such as time, temperature, and the ratio of raw material to solvent, which are crucial for the final quality of the extracts as well as the form of material preparation [18].

**Figure 1:**
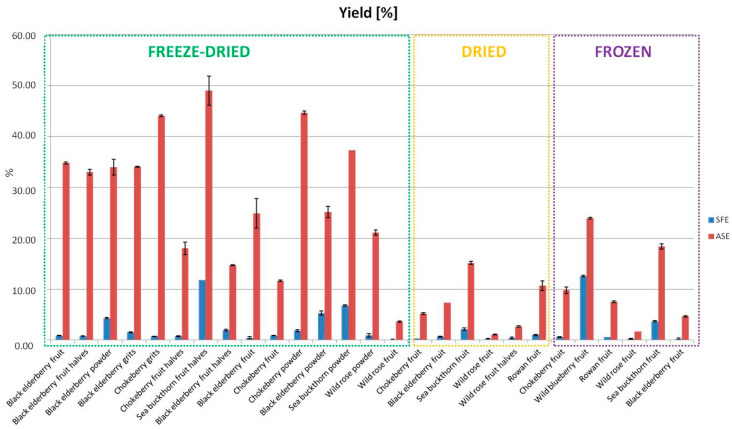
Extraction efficiency in extracts obtained by SFE and ASE.

The observed variations in extraction yield have significant implications for industrial scalability and cost-effectiveness. Higher yields, as demonstrated by the ASE method, are advantageous for industrial applications where maximizing output is critical to meeting demand and ensuring cost efficiency. Specifically, the ASE method achieved extraction yields exceeding 40% for certain materials, such as sea buckthorn fruit halves and chokeberry grits, compared to the lower yields (<10%) of SFE. This difference translates to lower raw material requirements and reduced processing time for ASE, directly reducing operational costs. However, the lower yields associated with SFE may be offset by the higher purity and stability of the extracts, which could justify the cost of high-value products like nutraceuticals. A more detailed economic analysis could evaluate whether the higher capital costs of ASE or SFE are mitigated by their respective efficiencies and product quality (Figure 1).

- Total phenolic content

**Figure 2:**
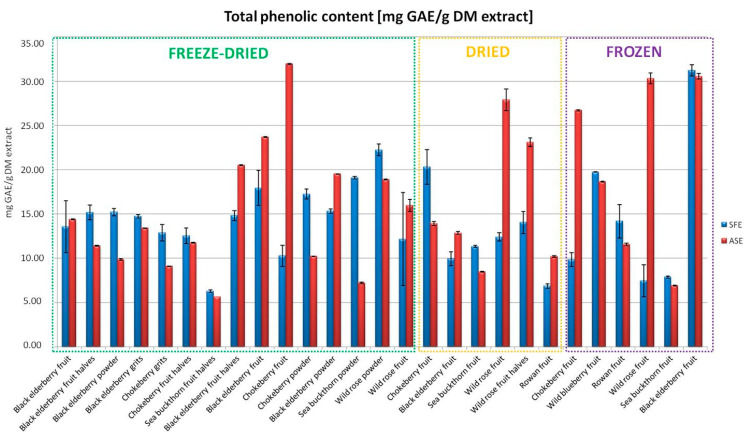
Total phenolic content in the obtained extracts using SFE and ASE.

The ASE method showed higher phenolic content values in most of the analyzed samples. The highest values were recorded for extracts from freeze-dried chokeberries (31.95 mg GAE/g of dry extract) and frozen fruits from wild rose (30.31 mg GAE/g of dry extract) and black elderberry (30.56 mg GAE/g of dry extract) (Figure 2). In the case of the SFE method, the highest phenolic values were obtained for frozen black elderberry fruits (31.23 mg GAE/g of dry extract) and freeze-dried powder from wild rose (22.25 mg GAE/g of dry extract) (Figure 2). To provide additional context, our findings were compared to the study by Patra et al. [17], where ASE achieved a 30–40% higher total phenolic content compared to traditional methods. In our study, the ASE method produced the highest phenolic content (e.g., 31.95 mg GAE/g of dry extract for freeze-dried chokeberries), which is approximately 20% higher than the SFE results for the same material. Similarly, the ASE extracts from frozen wild rose fruits (30.31 mg GAE/g) exceeded the phenolic content reported for Soxhlet extraction by Mustafa and Turner [19] by approximately 35%. These comparisons highlight the significant advantages of advanced extraction methods over conventional ones and validate their applicability in producing phenolic-rich extracts for the food industry.

- Carotenoid Content

**Figure 3:**
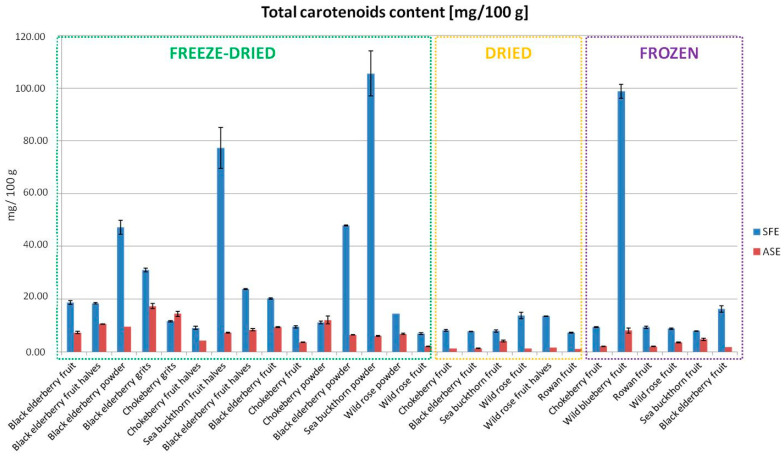
Total carotenoid content in extracts obtained using SFE and ASE.

Carotenoid stability is a critical factor influencing the effectiveness of extraction processes. Carotenoids are highly susceptible to oxidation and isomerization under heat, light, and oxygen exposure. The SFE method proved to be superior for carotenoid extraction, achieving significantly higher carotenoid content (e.g., 105.59 mg/100 g for freeze-dried sea buckthorn powder) compared to ASE (6.04 mg/100 g) (Figure 3). This can be attributed to the supercritical CO_2_ environment, which minimizes exposure to oxygen and operates at lower temperatures, preserving the bioactive compounds’ trans-isomeric forms. In contrast, the higher temperatures and pressures used in ASE might lead to partial degradation or isomerization of carotenoids. These findings underscore the importance of method selection in maintaining the structural integrity and bioactivity of carotenoids throughout the extraction process. These differences highlight the importance of selecting the extraction method based on the type of bioactive compounds we wish to obtain. The advantage of SFE arises from its ability to selectively extract low-polarity compounds, such as carotenoids, under supercritical conditions. Supercritical fluid extraction (SFE) prevents the oxidation of bioactive compounds during the extraction process, thereby helping to preserve the antioxidant activity of carotenoids by optimizing pressure and temperature, as well as improving the extraction of a more stable (trans) form of these compounds [20]. According to research by Mattea et al. [21], carotenoid extraction, particularly β-carotene, lutein, and lycopene, can be effectively carried out using supercritical carbon dioxide (CO_2_) technology. This extraction method stands out among other techniques because it provides better mass transfer. This is due to the low viscosity, surface tension, and density of supercritical CO_2_, which, combined with higher diffusion compared to conventional solvents, significantly enhances the efficiency of the extraction process [5,6].

To ensure the consistency of the results throughout the manuscript, we have added Table 2, which presents the total carotenoid content expressed per gram of dry mass extract. Additionally, these results confirm the superiority of the SFE method, as demonstrated by its significantly higher total carotenoid content compared to ASE across various plant materials.

- Antioxidant Activity (DPPH)

**Figure 4:**
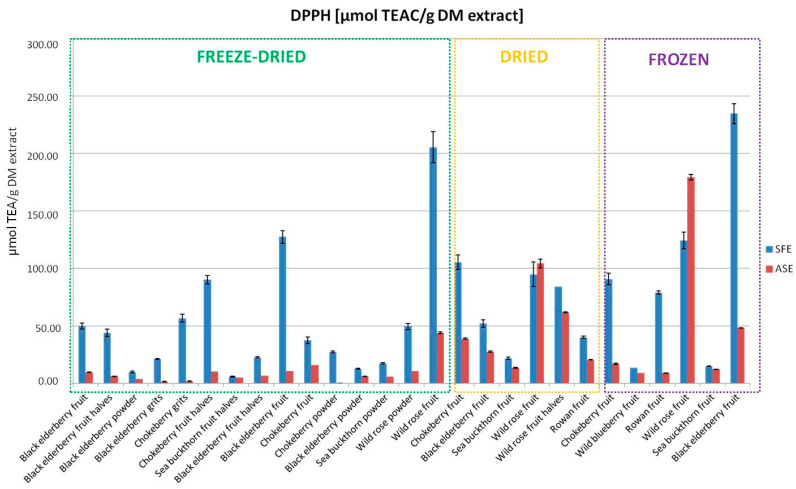
Antioxidant activity measured using the DPPH method in the extracts obtained using SFE and ASE.

The results of the DPPH test demonstrated varied antioxidant activity depending on the extraction method and type of fruit. The observed fluctuations in antioxidant activity across the 27 samples were analyzed using one-way ANOVA to determine the statistical significance between the two groups (ASE and SFE). It was determined that there were no statistically significant differences between the ASE and SFE methods for the extract from dried wild rose fruit. For all other samples, the differences in antioxidant activity between the two methods were found to be statistically significant. The highest DPPH values were obtained for extracts from frozen black elderberry fruits (234.67 µmol TEAC/g of dry extract) and freeze-dried wild rose powder (205.36 µmol TEAC/g of dry extract) using the SFE method (Figure 4). The ASE method showed lower values in the analyzed samples (Figure 4). These differences may arise from the varying abilities of the extraction methods to preserve and concentrate antioxidants. Antioxidant activity is closely related to the presence of phenols and carotenoids, suggesting that extraction methods should be tailored to specific goals to maximize health benefits. Significant differences in antioxidant potential may also result from the selectivity of the extraction processes. Paes et al. [22] also emphasize that while the SFE method can yield similar amounts of phenolic compounds as ASE, it produces extracts with higher antioxidant activity. The higher selectivity of SFE allows for better preservation of the bioactivity of phenols, which is particularly important given their role as antioxidants. SFE enables precise extraction of low-polarity compounds, which is advantageous for obtaining high-quality extracts. The results obtained may indicate a high level of interfering compounds in ASE extracts that do not exhibit biological activity. Shahid et al. [23] also note that while ASE is more efficient, it may lead to the extraction of interfering compounds that can diminish the quality and stability of final products. In contrast, extracts obtained using SFE, despite lower yields, exhibited high antioxidant potential, indicating greater selectivity of this technique. Therefore, extracts obtained by SFE may be particularly valuable for the food industry. SFE, due to supercritical conditions, allows for selective extraction of lipophilic compounds such as carotenoids, as confirmed by results obtained by Yaqoob et al. [24]. Carotenoids play a crucial role in antioxidant mechanisms, and their effective extraction is essential for achieving high antioxidant activity in industrial extracts. An additional advantage of this method is its ability to perform extraction without the addition of a co-solvent, making the final product ready for direct application in production. In the case of ASE extraction, evaporating the solvent can be problematic, requiring an additional step in the production process that involves investment in appropriate equipment as well as costs related to operation and electricity. In this study, the ASE method demonstrated lower antioxidant activity compared to SFE, which may be associated with the presence of these interfering compounds. These compounds can negatively affect the stability, clarity, and overall quality of extracts, representing a significant limitation of this technique.

### 3.2. Comparison of Different Methods of Processing Plant Material

Table 1 presents various forms of processed plant material selected for their health-promoting properties and potential in functional food production. The choice of raw materials was based on their ethnopharmacological and organoleptic characteristics, as well as their popularity among consumers, making them suitable candidates for further research on their application in innovative food products. The selection of processing methods for fruits is crucial for preserving their nutritional value and bioactive properties. Freeze-drying has proven to be the most effective method for maintaining bioactive compounds, while hot air drying often leads to significant losses of these compounds [25]. Freezing, although effective in maintaining quality, poses challenges in terms of storage. Understanding the impact of plant raw material preparation methods on the content of bioactive compounds, as well as potential issues related to their processing, is essential for the food industry striving to produce healthy and high-quality products. This knowledge allows for the optimization of technological processes to maximize the content of desired bioactive components in final products. Furthermore, identifying and addressing technological challenges associated with processing raw materials rich in bioactive compounds enables the development of efficient and effective methods for their extraction and stabilization. The aim of the study was to understand which of the most popular methods of preparing plant raw material—namely drying, freeze-drying, or freezing—best preserves the beneficial bioactive properties of the selected plant material.

- Freeze-drying

Freeze-drying demonstrated the highest efficiency in preserving bioactive compounds, as shown by quantitative data from this study. For example, extracts from freeze-dried chokeberry powder contained the highest total phenolic content (31.95 mg GAE/g using ASE), while for dried chokeberry powder was 13.93 mg GAE/g. In the case of SFE extracts, the highest levels of carotenoids were obtained for the extract from freeze-dried sea buckthorn powder (105.59 mg/100 g). Moreover, for the extract obtained by SFE from freeze-dried sea buckthorn fruit halves, the total carotenoid content was almost 10 times higher (77.39 mg/100 g) compared to the extracts from dried and frozen sea buckthorn fruit (7.86 mg/100 g). These differences can be attributed to the low temperatures used in freeze-drying, which minimize oxidative and thermal degradation. The results for black elderberry and sea buckthorn extracts in the form of freeze-dried powders are consistent with previous studies suggesting that freeze-drying can preserve up to 90% of phenols and antioxidant activity compared to fresh fruits. The obtained results confirm earlier literature findings that identify freeze-drying as the most effective method for maintaining bioactive compounds in fruits [26,27].

- Drying

Drying is currently one of the most common methods for preserving nutrients and bioactive compounds during food production [22]. Extracts obtained from dried berry fruits exhibited lower levels of bioactive compounds (Figure 1) and total carotenoid content (Figure 3). An exception was noted for extracts from whole wild rose fruits, where the level of phenolic compounds was higher than that in freeze-dried fruits. This difference may result from structural changes occurring during the drying process, which facilitate better mass transfer in larger fruit materials (1–2 cm). In the case of powders, tissue fragments are small in both dried and freeze-dried fruits, leading to effective mass transport in both forms. Therefore, additional structural changes that occur during drying do not significantly affect mass transfer [7,28].

- Frozen

Freezing demonstrated variable efficiency depending on the type of fruit, with frozen wild berries achieving results comparable to freeze-dried samples. Extracts from frozen fruits exhibited high levels of phenolic compounds. This is likely due to two factors: (i) the good preservation of phenolic acid structures and (ii) the structural changes that occur during freezing, which positively affect mass transfer. Numerous studies have shown that freezing typically preserves more bioactive compounds than traditional drying methods. For example, a study on strawberries found that freezing retained 90% of the vitamin C content compared to only 50% in air-dried samples [29]. It is important to note that extracting from frozen plant raw materials was problematic due to specific storage conditions, difficulties in filling extraction systems, and the presence of water in the resulting extract, which increases the risk of microbial contamination. Therefore, freeze-drying proved to be more advantageous than freezing in terms of preserving bioactive compounds in certain fruits and its suitability for use in the food processing industry.

### 3.3. Chemical Analysis of Obtained Extracts by HPLC-ESI-MS/MS

Analysis of HPLC conducted on extracts obtained through SFE and ASE methods revealed significant differences in the content of key bioactive compounds, such as flavone, hesperidin, and chlorogenic acid, depending on the extraction method used and the form of plant material processing (Table 3).

- Flavone

In the case of flavone, the SFE method demonstrated significantly greater efficiency for freeze-dried wild rose fruits, where the content of these compounds reached 275.6 µg/100 g. In comparison, the ASE method yielded only 129.1 µg/100 g of flavone from dried fruits of the same species. These results may indicate that supercritical CO_2_, particularly in combination with modifiers, better dissolves flavones due to its lower polarity and specific extraction properties.

- Hesperidin

Hesperidin, which is a more polar compound, was better extracted using ASE. The highest concentration (199.7 µg/100 g) achieved for frozen wild rose fruits may be associated with the better solubility of hesperidin in more polar solvents used in this method. In the SFE method, although relatively high values were obtained in some cases, such as for dried wild rose fruit halves (67.3 µg/100 g), overall extraction of hesperidin was less efficient, which may result from the limited ability of supercritical CO_2_ to solvate more polar compounds without appropriate modifiers.

- Chlorogenic Acid

The highest content of chlorogenic acid was obtained using SFE from freeze-dried chokeberry powder, reaching a value of 813.3 µg/100 g. Such a high concentration may indicate a strong selectivity of the SFE method for extracting this compound, especially when ethanol is used as a modifier. In comparison, the ASE method achieved the highest values of chlorogenic acid for frozen wild rose fruits (149.8 µg/100 g), which is significantly lower. This suggests that SFE may be a more effective extraction method for medium-polarity compounds like chlorogenic acid while preserving their chemical integrity.

Different forms of processing had a significant impact on extraction efficiency. Freeze-drying proved to be the best method for preserving bioactive compounds, which can be attributed to minimizing losses due to thermal and oxidative degradation. An example is freeze-dried chokeberry powder, which showed the highest concentration of chlorogenic acid (813.3 µg/100 g) using SFE, significantly surpassing other processing forms. Freezing also demonstrated high efficacy in preserving phenolic compounds; however, challenges related to storage and potential microbial contamination may limit its application in industry.

### 3.4. Selectivity of Extraction Methods

**Figure 5:**
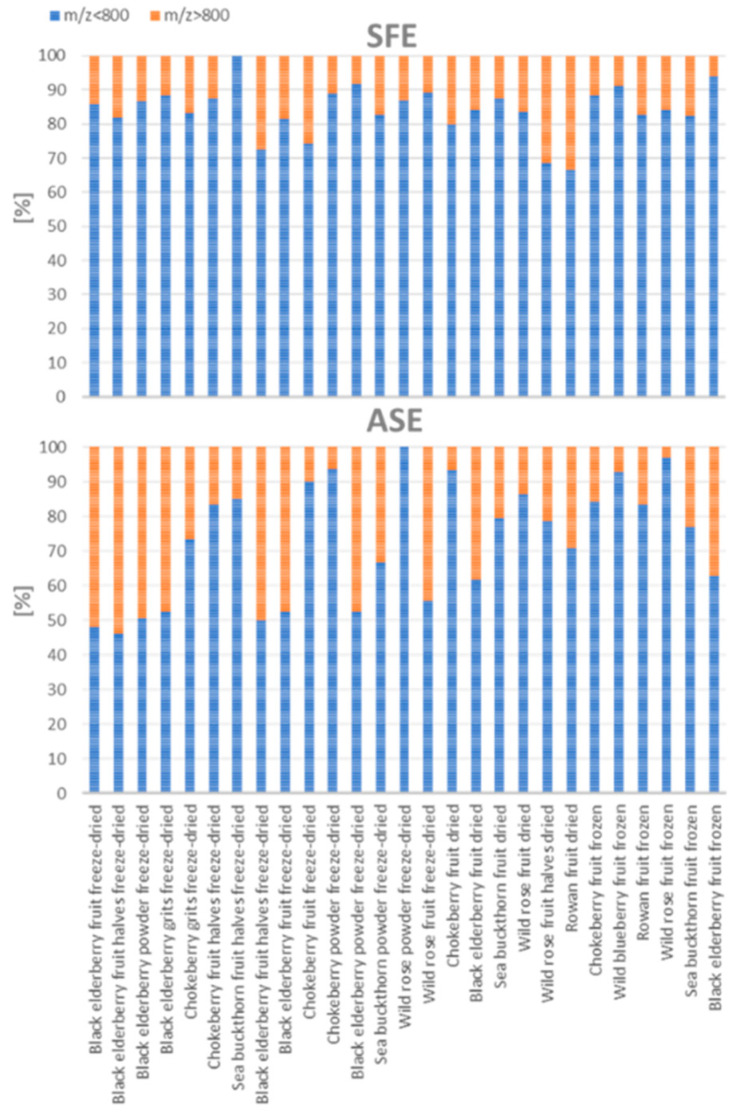
Percentage distribution of *m*/*z* values below and above 800 in plant extracts obtained using two extraction methods: accelerated solvent extraction (ASE) and supercritical fluid extraction (SFE).

**Figure 6:**
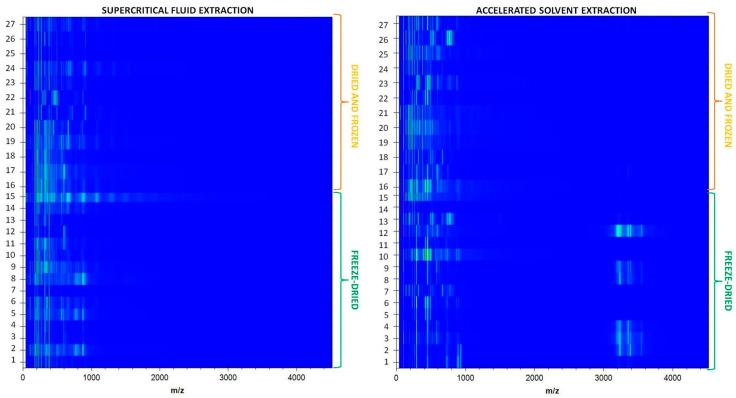
Gel view of MALDI-TOF-MS spectra in plant extracts (1–27) obtained using supercritical fluid extraction (SFE) and accelerated solvent extraction (ASE): 1. Black elderberry fruit freeze-dried, 2. Black elderberry fruit halves freeze-dried, 3. Black elderberry powder freeze-dried, 4. Black elderberry grits freeze-dried, 5. Chokeberry grits freeze-dried, 6. Chokeberry fruit halves freeze-dried, 7. Sea buckthorn fruit halves freeze-dried, 8. Black elderberry fruit halves freeze-dried, 9. Black elderberry fruit freeze-dried, 10. Chokeberry fruit freeze-dried, 11. Chokeberry powder freeze-dried, 12. Black elderberry powder freeze-dried, 13. Sea buckthorn powder freeze-dried, 14. Wild rose powder freeze-dried, 15. Wild rose fruit freeze-dried, 16. Chokeberry fruit dried, 17. Black elderberry fruit dried, 18. Sea buckthorn fruit dried, 19. Wild rose fruit dried, 20. Wild rose fruit halves dried, 21. Rowan fruit dried, 22. Chokeberry fruit frozen, 23. Wild blueberry fruit frozen, 24. Rowan fruit frozen, 25. Wild rose fruit frozen, 26. Sea buckthorn fruit frozen, 27. Black elderberry fruit frozen.

In this study, two extraction techniques—ASE and SFE—were compared in terms of their impact on the mass profile of the analyzed using MALDI-TOF-MS. The results indicate significant differences in the distribution of mass-to-charge ratios (*m*/*z*) between the extracts obtained using both methods (Figure 5 and Figure 6).

Figure 5 illustrates the percentage distribution of *m*/*z* (mass-to-charge ratio) values below and above 800 in extracts obtained from various plant materials using two extraction methods, ASE and SFE. The interpretation of this data focuses on comparing the efficiency and selectivity of these two extraction techniques in isolating compounds of different molecular weights. Firstly, the distinction between ASE and SFE methods may influence their efficiency in extracting molecules of different *m*/*z* ranges. SFE shows a higher percentage of *m*/*z* values below 800, which indicates that this method is more effective in isolating smaller molecules from plant materials. Conversely, ASE shows a greater proportion of *m*/*z* values above 800, which suggests that this method may extract larger compounds. Molecules with *m*/*z* values below 800 often correspond to smaller chemical molecules such as flavonoids or alkaloids, while those with *m*/*z* values above 800 may represent more complex structures, including interfering molecules like lipids or proteins. These findings are consistent with previous reports indicating that SFE is more effective in extracting small-molecule secondary metabolites, while ASE prefers the extraction of compounds with more complex molecular structures [7].

Additionally, the gel view presented in Figure 6 provides further insights into the differences in the chemical composition of the obtained extracts. The spectra obtained for samples from SFE and ASE show differences in signal intensity and distribution, suggesting that extraction methods influence the final chemical composition of the extracts. SFE exhibits a more uniform spectral profile for all tested extracts, with almost all *m*/*z* values below 1000, which may result from the greater selectivity of this method. In contrast, ASE, due to the use of higher temperatures and pressures, appears to facilitate the release of a more diverse range of higher molecular weight compounds, especially for freeze-dried and dried elderberry. Such differences may have significant implications for the applications of extracts in pharmacology and biotechnology, where specific classes of chemical compounds are preferred. From a practical standpoint, the choice of extraction method should depend on the intended goal of the study. If the aim is to obtain low molecular weight compounds such as terpenes or phenols, SFE seems to be the more appropriate technique. Conversely, ASE may be a better choice when higher molecular weight compounds such as alkaloids, polysaccharides, or proteins are desired. This indicates a potential complementarity between both techniques in studies on plant constituents.

## 4. Conclusions

The results of this study clearly demonstrate that ASE and SFE methods offer distinct advantages for extracting bioactive compounds. ASE is highly efficient, especially for phenolic compounds and antioxidants, making it ideal for maximizing yield. However, its efficiency may introduce interfering non-bioactive compounds, potentially reducing product quality. In contrast, SFE provides higher selectivity and better preservation of carotenoids and lipophilic compounds, producing purer extracts with minimal interference. Combining SFE with freeze-drying effectively preserves bioactive properties, as shown in studies on black elderberry and sea buckthorn, maintaining high phenol and carotenoid concentrations. This approach is particularly promising for high-quality berry fruit extracts, supporting innovations in functional foods.

Freezing and drying methods, however, lead to greater bioactive compound losses, limiting their application. Future research should optimize ASE and SFE parameters to improve efficiency, scalability, and compatibility with diverse plant materials. This includes exploring novel SFE co-solvents for polar compounds, refining ASE to protect heat-sensitive substances, and assessing the economic and environmental feasibility of industrial integration. Hybrid systems combining ASE and SFE could enhance yields and selectivity, unlocking new opportunities in functional foods and nutraceuticals.

## Figures and Tables

**Table 1:** Selected plant materials.

Producer	Plant Material	Form of Plant Material Processing
LIOGAM Foryś, Kot, Prześlak sp.J Kielce Poland	Black elderberry fruit	Freeze-dried
	Black elderberry fruit halves	Freeze-dried
	Black elderberry powder	Freeze-dried
	Black elderberry grits	Freeze-dried
WPPH “ELENA”, Kalisz, Poland	Chokeberry grits	Freeze-dried
	Chokeberry fruit halves	Freeze-dried
	Sea buckthorn fruit halves	Freeze-dried
	Black elderberry fruit halves	Freeze-dried
PPHU “FROSTER”, Kielce, Poland	Black elderberry fruit	Freeze-dried
	Chokeberry fruit	Freeze-dried
	Chokeberry powder	Freeze-dried
	Black elderberry powder	Freeze-dried
	Sea buckthorn powder	Freeze-dried
	Wild rose powder	Freeze-dried
	Wild rose fruit	Freeze-dried
PPHU “AWB” Alina Becia Łańcut, Poland	Chokeberry fruit	Dried
	Black elderberry fruit	Dried
	Sea buckthorn fruit	Dried
	Wild rose fruit	Dried
	Wild rose fruit halves	Dried
	Rowan fruit	Dried
FUNGOPOL, Sp. z o.o Sp.k. Brusy, Poland	Chokeberry fruit	Frozen
	Wild blueberry fruit	Frozen
	Rowan fruit	Frozen
	Wild rose fruit	Frozen
	Sea buckthorn fruit	Frozen
	Black elderberry fruit	Frozen

**Table 2:** Total carotenoid content in extracts obtained using SFE and ASE calculated per gram of dry mass of the extract.

		Total Carotenoids Content [mg/g DM Extract]	
Plant Material	Form of Plant Material Processing	SFE	ASE
Black elderberry fruit	Freeze-dried	21.09 ± 1.24	0.21 ± 0.02
Black elderberry fruit halves	Freeze-dried	23.25 ± 1.29	0.32 ± 0.01
Black elderberry powder	Freeze-dried	11.01 ± 0.42	0.28 ± 0.02
Black elderberry grits	Freeze-dried	20.64 ± 0.96	0.51 ± 0.04
Chokeberry grits	Freeze-dried	15.74 ± 1.78	0.33 ± 0.03
Chokeberry fruit halves	Freeze-dried	12.06 ± 0.73	0.23 ± 0.03
Sea buckthorn fruit halves	Freeze-dried	6.56 ± 0.93	0.15 ± 0.02
Black elderberry fruit halves	Freeze-dried	12.19 ± 1.20	0.57 ± 0.05
Black elderberry fruit	Freeze-dried	44.45 ± 3.03	0.37 ± 0.08
Chokeberry fruit	Freeze-dried	10.78 ± 0.10	0.31 ± 0.01
Chokeberry powder	Freeze-dried	5.95 ± 0.53	0.27 ± 0.05
Black elderberry powder	Freeze-dried	9.05 ± 1.01	0.26 ± 0.01
Sea buckthorn powder	Freeze-dried	15.55 ± 1.38	0.16 ± 0.01
Wild rose powder	Freeze-dried	15.05 ± 0.86	0.32 ± 0.01
Wild rose fruit	Freeze-dried	62.39 ± 4.13	0.56 ± 0.02
Chokeberry fruit	Dried	25.27 ± 1.32	0.23 ± 0.01
Black elderberry fruit	Dried	11.11 ± 1.83	0.18 ± 0.01
Sea buckthorn fruit	Dried	3.71 ± 0.96	0.27 ± 0.03
Wild rose fruit	Dried	49.20 ± 3.32	1.08 ± 0.14
Wild rose fruit halves	Dried	33.88 ± 2.13	0.57 ± 0.04
Rowan fruit	Dried	7.19 ± 1.45	0.10 ± 0.01
Chokeberry fruit	Frozen	15.60 ± 0.24	0.21 ± 0.01
Wild blueberry fruit	Frozen	46.24 ± 3.06	0.34 ± 0.05
Rowan fruit	Frozen	15.47 ± 1.53	0.27 ± 0.01
Wild rose fruit	Frozen	34.23 ± 2.26	2.05 ± 0.15
Sea buckthorn fruit	Frozen	2.11 ± 0.07	0.25 ± 0.02
Black elderberry fruit	Frozen	64.76 ± 3.61	0.37 ± 0.01

**Table 3:** Flavone, hesperidin, and chlorogenic acid content in extracts measured using HPLC-MS.

				SFE						ASE			
Plant Material	Form of Plant Material Processing	Flavone [µg/100 g]	SD	Hesperidin [µg/100 g]	SD	Chlorogenic Acid [µg/100 g]	SD	Flavone [µg/100 g]	SD	Hesperidin [µg/100 g]	SD	Chlorogenic Acid [µg/100 g]	SD
Black elderberry fruit	Freeze-dried	12.1	2.3	3.5	0.7	236.7	35.8	5.1	4.8	8.3	4.5	20.3	0.6
Black elderberry fruit halves	Freeze-dried	6.1	0.3	3.6	0.4	111.5	0.0	5	0.2	10.6	1.2	16.6	1.2
Black elderberry powder	Freeze-dried	2.4	0.3	1.2	0.1	ND	ND	3.8	0.3	6.3	0.2	5.5	0.2
Black elderberry grits	Freeze-dried	8.9	0.7	4.6	0.8	222.3	17.4	4.4	0.2	7.5	0.5	24.4	0.3
Chokeberry grits	Freeze-dried	7.8	0.3	7.4	0.7	249.0	35.4	3.2	0.0	13.6	0.1	33.1	0.4
Chokeberry fruit halves	Freeze-dried	22.5	2.1	5.1	0.1	ND	ND	6.2	0.1	29.9	0.3	41.4	0.0
Sea buckthorn fruit halves	Freeze-dried	23.6	1.1	11.0	0.2	ND	ND	4.1	0.8	9.3	0.2	0.1	0.0
Black elderberry fruit halves	Freeze-dried	13.2	3.8	3.4	0.1	202.4	1.0	8.8	0.2	16.7	0.4	48.7	0.0
Black elderberry fruit	Freeze-dried	32.7	1.1	3.4	0.5	ND	ND	8.4	0.3	14.3	0.7	15.6	0.0
Chokeberry fruit	Freeze-dried	25.1	0.3	6.6	0.1	98.0	4.0	14	0.7	18.9	1.6	84	0.8
Chokeberry powder	Freeze-dried	21.4	0.3	9.5	0.0	813.3	30.0	3.5	0.0	31.1	0.8	61.8	1.8
Black elderberry powder	Freeze-dried	15.7	0.0	1.9	0.0	63.8	7.6	3.6	0.3	77.1	0.0	11.6	0.8
Sea buckthorn powder	Freeze-dried	30.4	2.1	1	0.0	32.1	0.4	3.3	0.0	0.2	0.0	0.2	0.0
Wild rose powder	Freeze-dried	21.8	1.1	ND	ND	86.0	3.1	6.9	0.5	8.9	0.1	0.8	0.0
Wild rose fruit	Freeze-dried	275.6	32.6	ND	ND	ND	ND	33.6	0.2	14.4	0.6	6.2	0.1
Chokeberry fruit	Dried	61.5	2.7	17.8	0.6	ND	ND	22.8	0.0	3.4	0.6	4.0	0.0
Black elderberry fruit	Dried	50.0	1.4	43.3	1.7	ND	ND	14.7	0.2	2.7	0.0	ND	ND
Sea buckthorn fruit	Dried	22.7	1.8	26.9	2.0	0.6	0.0	7.1	0.1	1.3	0.0	1.5	0.0
Wild rose fruit	Dried	38.6	0.4	19.3	0.5	ND	ND	129.1	13.0	22.8	0.8	ND	ND
Wild rose fruit halves	Dried	40.9	1.6	67.3	0.8	ND	ND	61.1	3.2	26.0	0.5	ND	ND
Rowan fruit	Dried	29.2	0.9	11.0	0.3	10.2	4.7	19.4	2.2	21.3	0.0	1.5	0.1
Chokeberry fruit	Frozen	29.4	4.7	ND	ND	596.0	35.7	10.3	1.2	2.1	0.0	23.5	1.1
Wild blueberry fruit	Frozen	0.9	0.0	14.7	2.0	30.1	1.0	5.2	0.0	62.1	4.6	138.4	0.5
Rowan fruit	Frozen	35.3	3.0	14.3	0.6	ND	ND	13.2	1.1	25.4	1.1	ND	ND
Wild rose fruit	Frozen	32.3	3.0	21.3	2.6	6.6	0.1	117.1	9.8	199.7	15.1	149.8	5.3
Sea buckthorn fruit	Frozen	22.7	1.1	21.4	1.1	ND	ND	6.2	0.4	2.8	0.1	1.0	0.1
Black elderberry fruit	Frozen	81.1	7.9	65.1	5.2	ND	ND	24.6	1.5	12.4	1.4	ND	ND

ND—not detected; SD—standard deviation.

## Data Availability

The original contributions presented in the study are included in the article; further inquiries can be directed to the corresponding author.
